# Turmeric-Containing Polymer–Mineral Composites with a Waste Cooking Oil-Derived Binder: Physicochemical Characterisation and Exploratory Antimicrobial Screening

**DOI:** 10.3390/ma19173583

**Published:** 2026-08-24

**Authors:** Anita Zawadzka, Magda Kijania-Kontak, Barbara Pucelik, Agata Barzowska-Gogola, Mateusz Barczewski, Sandra Paszkiewicz, Zbigniew Rozwadowski, Paweł Staroń

**Affiliations:** 1 Department of Engineering and Chemical Technology, Cracow University of Technology, 24 Warszawska St., 31-155 Cracow, Poland; pawel.staron@pk.edu.pl; 2 Department of Civil Engineering, Cracow University of Technology, 24 Warszawska St., 31-155 Cracow, Poland; magda.kijania-kontak@pk.edu.pl; 3 Łukasiewicz Research Network, Cracow Institute of Technology, 73 Zakopianska St., 30-418 Cracow, Polandagata.barzowska-gogola@kit.lukasiewicz.gov.pl (A.B.-G.); 4 Institute of Materials Technology, Poznan University of Technology, 3 Piotrowo St., 61-138 Poznan, Poland; 5 Department of Materials Technologies, West Pomeranian University of Technology, 17 Av. Piastow, 70-310 Szczecin, Poland; sandra.paszkiewicz@zut.edu.pl; 6 Department of Inorganic and Analytical Chemistry, West Pomeranian University of Technology, 17 Piastow Av., 70-310 Szczecin, Poland

**Keywords:** waste cooking oil, oil-derived binder, polymer–mineral composite, turmeric-modification, thermal curing, hydrophobicity, antimicrobial screening, circular materials

## Abstract

Waste cooking oil (WCO) was investigated as a waste-derived reactive precursor for a cured organic binder in highly mineral-filled composites containing turmeric. Ten formulations selected from a broader experimental screening were prepared from WCO, sulfuric acid, quartz sand, and turmeric added at 1–7% relative to the dry mass of quartz sand. Formulation-specific thermal curing was conducted at 190–210 °C for 12–20 h. Because the binder content, acid-to-binder ratio, turmeric content, curing temperature, and curing time varied simultaneously, the study was designed as an exploratory multifactorial screening rather than as a controlled assessment of individual processing variables. FTIR, ^1^H NMR analysis of acetone-soluble constituents, TGA, and SEM-EDS were combined with mechanical screening, water-absorption, contact-angle, and microbiological measurements. Bending and splitting tensile strengths ranged from 1.15 to 2.42 MPa and from 0.30 to 0.52 MPa, respectively. Water absorption ranged from approximately 4.8% to 9.0%, while water contact angles exceeded 90° for all investigated formulations. Selected formulations reduced the surviving fraction by more than 90% for *Staphylococcus epidermidis* and by approximately 70% for *Pseudomonas aeruginosa* under the applied suspension-assay conditions. The estimated C_50_ values of 60.6–83.3 mg mL^−1^ indicated measurable concentration-dependent responses at relatively high nominal composite concentrations. The available results support curing-associated transformation and consolidation of the WCO-derived binder phase but do not quantify crosslink density or retained organic content. Because no matched turmeric-free composite or surface/eluate pH measurements were available, the biological effects are attributed to the complete composite formulations rather than specifically to turmeric or intact curcumin. The results provide an exploratory basis for the further development of waste-derived, non-load-bearing polymer–mineral composites with functional surface and biological properties.

## 1. Introduction

Global vegetable-oil production reached approximately 224.2 million metric tons in the 2024/2025 season, reflecting the continuing growth in the consumption and processing of vegetable oils [1]. This trend is accompanied by the generation of substantial quantities of waste cooking oil (WCO). Reported estimates indicate approximately 3.7 billion gallons of used cooking oil generated in 2022, with projected volumes of 5–10 billion gallons by 2030 [2]. In Europe, WCO generation has been estimated at approximately 8 L per capita annually [3]. Improper disposal of WCO may contribute to wastewater-system problems and environmental contamination, whereas its conventional valorisation remains dominated by fuel-oriented routes. Consequently, alternative pathways that convert WCO into stable, value-added materials are receiving increasing attention [3,4]. Previous studies have demonstrated the feasibility of incorporating WCO into mineral-filled and construction-related composite materials, supporting its potential as a waste-derived precursor for non-conventional material systems [4,5].

Waste cooking oil (WCO) is a chemically heterogeneous lipid waste generated after repeated thermal and oxidative exposure during food preparation [6]. Its conversion into higher-value materials is increasingly investigated as an alternative to disposal or exclusive fuel-oriented use [7,8,9]. Vegetable oils and WCO can act as renewable or waste-derived precursors for polymeric binders and filled composites; previous studies have reported WCO-containing roofing tiles and other WCO-based composite materials [5]. Their triglyceride structures contain ester groups and unsaturated fatty-acid chains that may participate in further reactions during chemical modification and thermal treatment.

In mineral-filled WCO composites, the oil-derived phase must undergo sufficient chemical and physical transformation to immobilise the liquid feedstock and bind the filler. WCO is susceptible to esterification and transesterification reactions [10,11], while thermal curing of an unsaturated lipid phase may also involve oxidation, oligomerisation and reactions at unsaturated sites. Because several transformations may occur concurrently, spectroscopic changes and macroscopic solidification can be interpreted as evidence of curing-associated transformation, but not as direct quantification of crosslink density. Dedicated measurements such as solvent extraction, gel-fraction analysis, swelling experiments or dynamic mechanical analysis would be required to establish network density, particularly in highly filled systems in which the organic binder is a minority phase. In the present work, the term “polymer–mineral composite” is used to describe a highly mineral-filled material in which the thermally transformed WCO-derived organic phase acts as a solid binder consolidating the quartz particles. This terminology does not imply that the formation, chemical structure, or density of a covalently crosslinked network was directly established. The polymer-related aspect of the study concerns the chemical and physical transformation of the lipid-derived precursor into a cured organic binder phase and its formulation-level modification with a plant-derived additive.

Plant-derived modifiers may introduce additional surface chemistry into such composites. Turmeric (*Curcuma longa* L.) contains curcuminoids and other aromatic constituents with reported antibacterial and antifungal activity [4,12,13,14,15]. However, because the composites were cured for 12–20 h at 190–210 °C, the persistence of intact curcumin cannot be assumed without compound-specific quantification. The cured WCO/acid-containing phase, residual acidity, surface wettability, porosity and thermally transformed turmeric constituents may all contribute to the measured biological response.

Previous studies on WCO-based composites have addressed material production and mechanical or water-related properties [5,16], environmental interactions [4], and antimicrobial modification using hops and curly sorrel [17]. However, the integrated characterisation of turmeric-containing WCO-based mineral composites across structural, thermal, mechanical, surface and microbiological endpoints remains limited. The research gap addressed in this work is the limited understanding of how formulation-level modification with a plant-derived additive and thermal processing are reflected simultaneously in the structural, thermal, mechanical, surface, and biological response of mineral-filled composites containing a WCO-derived cured binder. The novelty of the study lies in the integrated evaluation of these property domains within the same set of turmeric-containing formulations. Importantly, the study distinguishes directly measured material properties from proposed chemical or antimicrobial mechanisms that could not be independently verified using the available experimental design.

Accordingly, the objective of this study was to characterise ten turmeric-containing WCO-based polymer-mineral formulations selected from a broader screening set. The study examined their cured-state structure, thermal behaviour, morphology, mechanical screening response, water absorption, wettability and antimicrobial performance. Because the selected formulations differed simultaneously in binder content, acid-to-binder ratio, turmeric content, curing temperature and curing time, the work was not designed to isolate the independent effect of any single variable. Similarly, because an otherwise identical turmeric-free control was not included, the biological data are interpreted as formulation-level screening results and not as proof of turmeric-specific antimicrobial activity.

## 2. Materials and Methods

### 2.1. Preparation of Turmeric-Containing WCO-Based Polymer–Mineral Composites

The composites were prepared from post-consumer waste cooking oil (WCO), sulfuric acid (H_2_SO_4_), quartz sand, and powdered turmeric (*Curcuma longa* L.). The WCO consisted of rapeseed oil collected after food frying. It was filtered to remove visible solid residues and used without further purification. No additional physicochemical characterisation of the WCO was performed in the present study. Quartz sand with a particle-size range of 0.5–1.4 mm was used in the air-dried state. In this work, the term “catalysed oil” denotes the mixture of WCO and sulfuric acid. The contents of catalysed oil and turmeric reported in Table 1 were calculated relative to the dry mass of quartz sand and describe the nominal pre-curing formulations. These values should not be interpreted as measured post-curing mass fractions, because component-specific mass retention during thermal curing was not determined. For each batch, the combined dry mass of quartz sand and turmeric was 530 g. The components were mixed at 25 °C using an auto-mortar mixer (Matest, Arcore, Italy). WCO and sulfuric acid were first mixed for 3 min. Quartz sand was then added and mixed for 5 min, followed by turmeric and a further 3 min of homogenisation. The mixture was transferred into aluminium moulds for rectangular specimens (nominal mould dimensions, 25 × 20 × 100 mm) and cylindrical specimens (96 mm diameter and 24 mm height). An equal mass of the mixture was placed in each mould, and the filled moulds were compacted on a vibrating table. The specimens were thermally cured in laboratory oven SLN 115 SMART, Pol-Eko Aparatura company (Wodzislaw Slaski, Poland) under ambient-air conditions, using the formulation-specific temperature and time combinations listed in Table 1 (190–210 °C for 12–20 h). After curing, the specimens were removed from the moulds and conditioned at room temperature (25 °C) for 24 h before further testing. A total of 100 specimens were prepared. Ten formulations that produced mechanically intact specimens during preliminary screening and covered the investigated formulation and curing ranges were selected for further physicochemical and biological characterisation. The selected set was not a full factorial design and did not contain controlled one-variable-at-a-time series; accordingly, comparisons among formulations were treated as descriptive, and no independent causal effect was assigned to catalysed-oil content, acid-to-oil ratio, turmeric content, curing temperature, or curing time. An otherwise identical turmeric-free control composite was not prepared in this experimental series.

### 2.2. Physicochemical, Thermal, Morphological and Mechanical Characterisation

Fourier-transform infrared (FTIR) spectra were acquired in attenuated total-reflectance (ATR) mode using a Nicolet iS5 spectrometer (Thermo Scientific, Waltham, MA, USA) equipped with a diamond ATR accessory. Absorbance spectra were recorded over the range of 400–4000 cm^−1^ at a spectral resolution of 4 cm^−1^.

Proton nuclear magnetic resonance (^1^H NMR) spectroscopy was performed using a 400 MHz spectrometer (Bruker, Karlsruhe, Germany). A 0.1 g portion of composite was placed in a screw-cap vial, followed by the addition of 0.8 mL of deuterated acetone (acetone-d_6_ containing 0.03% TMS, 99.9 atom% D; CAS 666-52-4; Pol-Aura Sp. z o.o., Zawroty, Poland). The mixture was gently shaken for approximately 5 min and then left undisturbed at room temperature for approximately 4 h. The acetone phase was subsequently transferred into an NMR tube. Tetramethylsilane was used as the internal reference for chemical-shift calibration.

Thermogravimetric analysis (TGA) was performed for composite 1 using a Netzsch TG 209 F1 Libra^®^ instrument (Selb, Germany). An analytical portion of 10 ± 0.5 mg was placed in an Al_2_O_3_ pan and heated from 30 to 900 °C at 10 °C min^−1^ under nitrogen flowing at 50 mL min^−1^.

Composite morphology and elemental composition were examined using a Hitachi TM-3000 scanning electron microscope (Tokyo, Japan) coupled with an energy-dispersive X-ray spectrometer (EDS). Before analysis, the specimens were sputter-coated with a gold/palladium layer.

Mechanical testing was performed using a Zwick-Roell Z600 universal testing machine (Ulm, Germany). Flexural strength was determined in a three-point bending configuration based on PN-EN 12390-5 [18]. Rectangular specimens prepared in nominal 25 × 20 × 100 mm moulds were placed on supports with a span of 80 mm. After application of an initial load of 25 N, the load was increased at 5 N/s until specimen failure. The maximum force and corresponding deflection were recorded, and flexural strength was calculated according to the equation specified in PN-EN 12390-5. Splitting tensile tests were conducted on the cubic specimens using the same universal testing machine. After application of an initial load of 25 N, the load was increased at 10 N/s until specimen failure. Cubic specimens were prepared in moulds with nominal dimensions of 50 × 50 × 35 mm. The maximum splitting tensile strength and corresponding displacement were recorded. Mechanical tests were performed using 3 independently prepared specimens per formulation. Results are presented as mean values ± standard deviations.

Water absorption was determined using three replicate specimens for each formulation. Approximately 5 g specimens were obtained as fragments from the cured moulded composites and therefore contained both mould-formed and newly exposed surfaces. The fragments were selected to obtain comparable specimen masses across all formulations and were tested using the same preparation procedure. Each specimen was immersed for 72 h at 25 °C in a volume of water equal to 20 times its initial mass. After removal from water, the surface was gently blotted with lint-free paper and the specimen was weighed within 1 min to limit evaporation. Water absorption was calculated as WA (%) = [(m_72h_ − m_0_)/m_0_] × 100, where m_0_ is the initial specimen mass before immersion and m_72h_ is the mass after 72 h of immersion. Accordingly, the reported values should be interpreted as comparative water-uptake measurements for specimens prepared in this manner rather than as water absorption of intact moulded components.

Static contact angles were measured by sessile-drop imaging. The composite was placed on a horizontal surface, and a 50 µL droplet of deionised water or rapeseed oil was gently deposited on the specimen surface. The camera lens was aligned with the height of the composite surface, and side-view images of the droplet were acquired immediately after deposition, without an intentional waiting period. For image analysis, a baseline tangent to the flat composite surface and a tangent to the droplet profile were defined. The contact angle (θ) was measured through the liquid between the baseline and the tangent to the droplet profile using the Contact Angle plug-in. Angles above 90° were classified as hydrophobic for water and oleophobic for rapeseed oil. For each formulation and test liquid, measurements were performed at three independently selected surface locations. The results are presented as mean values ± SD.

### 2.3. Microbiological Testing

#### 2.3.1. Bacterial Strains and Culture Conditions

The following bacterial strains were used: Staphylococcus aureus 8325-4, *Staphylococcus epidermidis* ATCC 14990, *Escherichia coli* K12, and *Pseudomonas aeruginosa* ATCC 19660. *E. coli* and *P. aeruginosa* were cultured in LB broth, whereas *S. aureus* and *S. epidermidis* were cultured in brain heart infusion (BHI) broth. Stock cultures were stored at −80 °C in the corresponding liquid growth medium supplemented with 20% glycerol. Before each experiment, the bacteria were streaked onto the corresponding solid growth medium and incubated at 37 °C for 1–2 days. Individual bacterial colonies were subsequently inoculated into the corresponding liquid medium, and the precultures were incubated for 24 h at 37 °C with continuous shaking at 130 rpm. The optical density of the cultures was measured at 600 nm. Based on preliminary growth-curve measurements, an OD_600_ of 0.5 corresponded to the late logarithmic growth phase and was therefore used to standardise the bacterial cultures for the antibacterial experiments. The procedure was treated as a suspension-based screening assay and was not designated as an ISO 22196 test [19].

#### 2.3.2. Suspension-Based Antibacterial Assay

After approximately 24 h of growth in liquid medium, the bacterial cultures were centrifuged for 10 min at 4500 rpm. The supernatant was removed, and the bacterial pellet was resuspended in phosphate-buffered saline (PBS) to obtain an OD_600_ of 0.5. Viable plate-count enumeration confirmed that this optical density corresponded to approximately 1 × 10^8^ CFU mL^−1^ under the applied experimental conditions. For each exposure condition, 20 mg of the cured composite, mechanically ground prior to microbiological testing, was placed in a test vessel, and 200 µL of the prepared bacterial suspension was added. The ground composite was not subjected to controlled particle-size fractionation, and its specific or exposed surface area was not determined. Accordingly, the exposure was normalised on a mass-to-liquid-volume basis rather than on a surface-area basis. This corresponded to a nominal solid-material-to-liquid ratio of 100 mg mL^−1^. The term “nominal composite concentration” refers to the mass of solid composite material per volume of bacterial suspension and does not represent a dissolved concentration of an antimicrobial compound.

The bacterial suspensions were incubated in the presence of the composite material for 24 h under continuous mixing using a microplate shaker. After incubation, aliquots of the bacterial suspensions were subjected to serial ten-fold dilutions in PBS. Subsequently, 15 µL portions of the appropriate dilutions were plated onto the corresponding solid growth medium. The plates were incubated for a further 24 h at 37 °C, after which the bacterial colonies were counted and the number of colony-forming units per unit volume was calculated. The surviving fraction was expressed as a percentage relative to the corresponding untreated bacterial control. Measurements were performed in triplicate, and the results are presented as mean values ± SEM.

#### 2.3.3. Concentration-Response Analysis

For selected formulation–bacterium combinations that showed activity during the initial screening, concentration-dependent responses were additionally evaluated over the investigated nominal composite-concentration range. The surviving fraction was calculated relative to the corresponding untreated bacterial control and plotted as a function of nominal composite concentration. The concentration-response data were analysed in GraphPad Prism (GraphPad Software, LLC., Boston, MA, USA; https://www.graphpad.com/; accessed 21 April 2024) using nonlinear regression with an empirical sigmoidal fit. The fit was used solely as a descriptive interpolation procedure to estimate the nominal composite concentration corresponding to a surviving fraction of 50%, defined here as the estimated C_50_. The C_50_ estimates were not used for inferential comparisons among formulations. Measurements were performed in triplicate, and the experimental data are presented as mean values ± SEM.

#### 2.3.4. Confocal Microscopy of Selected Bacterial Biofilms

Selected bacterial biofilms were additionally examined by laser scanning confocal microscopy (Carl Zeiss Ltd., Jena, Germany). Bacterial colonies grown overnight on the corresponding agar medium were suspended in liquid medium to obtain an optical density of OD_490_ = 0.65. The suspension was diluted 1:6 and incubated at 37 °C in an atmosphere containing 5% CO_2_ for approximately 3 h to obtain the mid-logarithmic growth phase. The resulting suspension was subsequently diluted 1:2500 in pre-warmed growth medium, and 200 µL was transferred into each well of an eight-well plate. The plates were incubated at 37 °C in 5% CO_2_. After approximately 16 h, the growth medium was replaced with fresh medium, and the selected composite materials were added. The biofilms were exposed to the materials for 24 h and subsequently stained with propidium iodide, Calcein AM, and Hoechst dye. Untreated control biofilms were prepared, stained and imaged in parallel with the composite-exposed samples under the same experimental conditions. Propidium iodide and Calcein AM were used to visualise membrane-damaged and viable cells, respectively, whereas Hoechst dye was used for nucleoid staining. The samples were imaged by laser scanning confocal microscopy, and the acquired images were processed using Zeiss ZEN software (ZEISS; https://www.zeiss.com/microscopy/en/products/software/zeiss-zen.html; accessed 28 April 2024). The microscopy images were interpreted qualitatively; no quantitative image analysis was performed.

#### 2.3.5. Antifungal Screening

The antifungal response of the selected composite formulations was evaluated against *Pichia anomala* and *Aspergillus niger* in planktonic and biofilm forms. *P. anomala* cultures were maintained on Sabouraud dextrose agar and transferred onto freshly prepared agar plates, which were incubated at 30 °C for at least 48 h. Yeast extract-peptone-dextrose broth, Sabouraud dextrose broth and RPMI 1640 were used as growth media during the planktonic and biofilm assays. *A. niger* was inoculated into Czapek-peptone medium and cultured at 30 °C under orbital shaking at 100 rpm for at least 7 days before the experiments. The microorganisms were exposed to the selected composite formulations in PBS at a nominal material concentration of 100 mg mL^−1^ for 24 h in the dark at room temperature. After exposure, 10 µL aliquots were transferred to 1 mL of fresh growth medium in 12-well plates. The samples were mixed, serially diluted in PBS and plated for colony-forming unit enumeration. The surviving fraction was calculated from CFU counts and expressed as a percentage of the corresponding untreated microorganism control. Measurements were performed using three replicate samples per condition, and the results are presented as mean values ± SEM.

### 2.4. Statistical Treatment

The study was designed as an exploratory formulation-level screening rather than as a balanced factorial experiment. Because the selected formulations differed simultaneously in catalysed-oil content, H_2_SO_4_-to-catalysed-oil ratio, turmeric content, curing temperature and curing time, inferential comparisons among complete formulations cannot isolate the independent effect of any single formulation variable. Accordingly, statistical comparisons among formulations were not used to assign causal effects to individual composition or processing parameters.

Mechanical, water-absorption and contact-angle data are reported as mean values ± SD, whereas antibacterial-screening, concentration-response, and antifungal data are reported as mean values ± SEM, as specified in the corresponding methods and figure captions. For the microbiological screening data, each composite-exposed condition was compared with the corresponding untreated microbial control. The estimated C_50_ values obtained from the exploratory concentration-response curves were treated as descriptive estimates and were not used for hypothesis testing or for statistical ranking of the investigated formulations. For each microorganism and experimental condition, data were analysed separately using one-way analysis of variance (ANOVA) followed by Dunnett’s multiple-comparison test, with the untreated condition used as the reference group. A value of *p* < 0.05 was considered statistically significant. The untreated controls were used as reference groups for the statistical comparisons but are not displayed as separate categories because surviving fractions are expressed relative to the corresponding untreated controls.

These statistical comparisons were used only to determine whether exposure to a complete composite formulation produced a statistically significant response relative to the untreated control; they were not used to infer the independent contribution of turmeric or any other individual formulation variable. Nonlinear regression was used to estimate C_50_ values from the concentration-response curves. Confocal-microscopy images were interpreted qualitatively and were not included in inferential statistical comparisons.

## 3. Results and Discussion

### 3.1. Structural, Thermal, Morphological, Mechanical and Surface Characterisation of WCO-Based Composites

**Figure 1:**
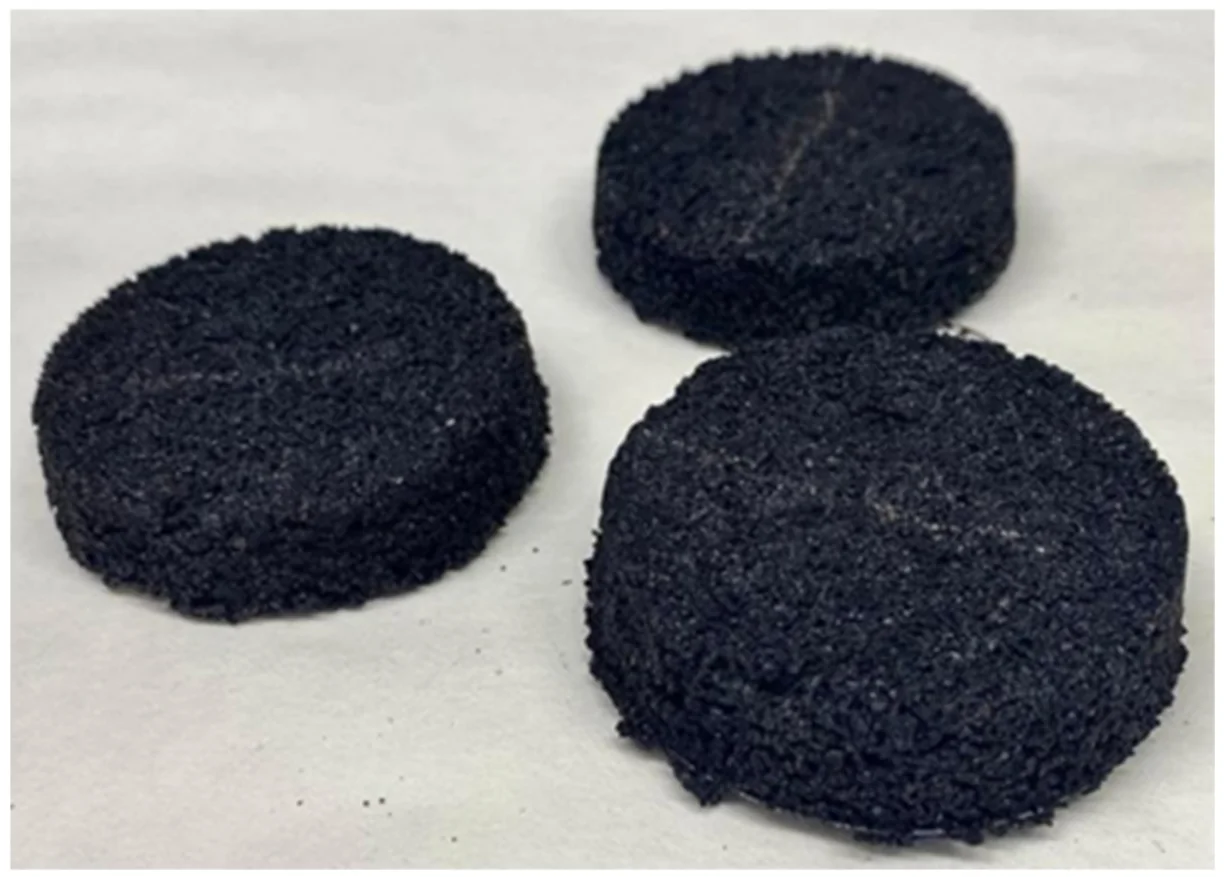
Photograph of composite with turmeric (No. 9).

The selected WCO-based formulations formed solid, porous, dark-brown specimens. This macroscopic solidification indicates that the initially liquid WCO-containing phase was converted into a cured binder capable of consolidating the quartz filler. However, visual inspection alone does not establish the formation, chemical structure or crosslink density of a polymeric network. An example of a turmeric-containing specimen is presented in Figure 1.

**Figure 2:**
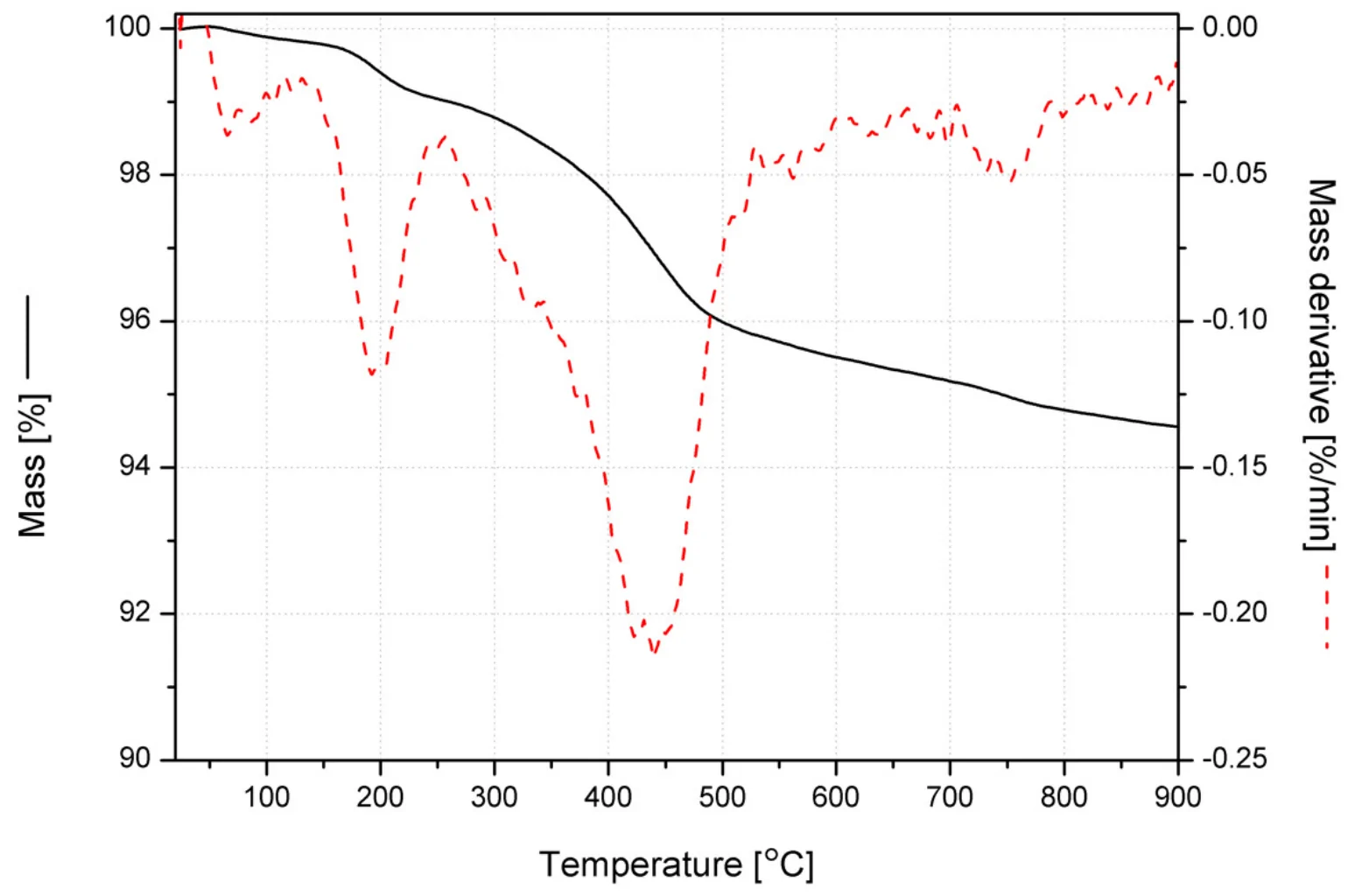
TGA and DTG curves of composite 1.

The TGA/DTG curve of sample 1 is presented in Figure 2. A small mass loss below approximately 100 °C, with a derivative maximum at 65.5 °C and a maximum mass-loss rate of −0.04% min^−1^, is consistent with the removal of physically adsorbed moisture [20]. A further minor event was observed around 192.3 °C, with a maximum mass-loss rate of −0.12% min^−1^, and may be associated with low-molecular-weight or thermally labile constituents. The principal mass-loss region extended from approximately 250 to 600 °C, with a maximum rate at 439.6 °C (−0.22% min^−1^), and is assigned broadly to thermal decomposition of the cured organic fraction [21,22]. For sample 1, the mass remaining at 900 °C was 94.56% under nitrogen. This value is reported only as the residue obtained for the analysed TGA portion and should not be interpreted as the mineral mass fraction or as a post-curing mass balance. The nominal catalysed-oil addition reported in Table 1 describes the pre-curing formulation, whereas the final TGA residue under nitrogen may contain quartz, sulfur-containing residues, turmeric-derived ash, and carbonaceous char formed during pyrolysis. Moreover, the 10 ± 0.5 mg analytical mass is small relative to the 0.5–1.4 mm quartz particle size. Consequently, local variation in the mineral-to-organic ratio within the analysed portion cannot be excluded, and its representativeness of bulk composite 1 cannot be assured. The discrepancy between the nominal formulation and the observed TGA mass loss may therefore reflect sampling heterogeneity, curing-related mass loss and/or char formation during the nitrogen TGA experiment; the relative contribution of these effects cannot be resolved from the available data. Accordingly, the retained organic fraction cannot be calculated from the present TGA curve. Because TGA was performed only for formulation 1, these observations are not generalised to the other composite formulations.

**Figure 3:**
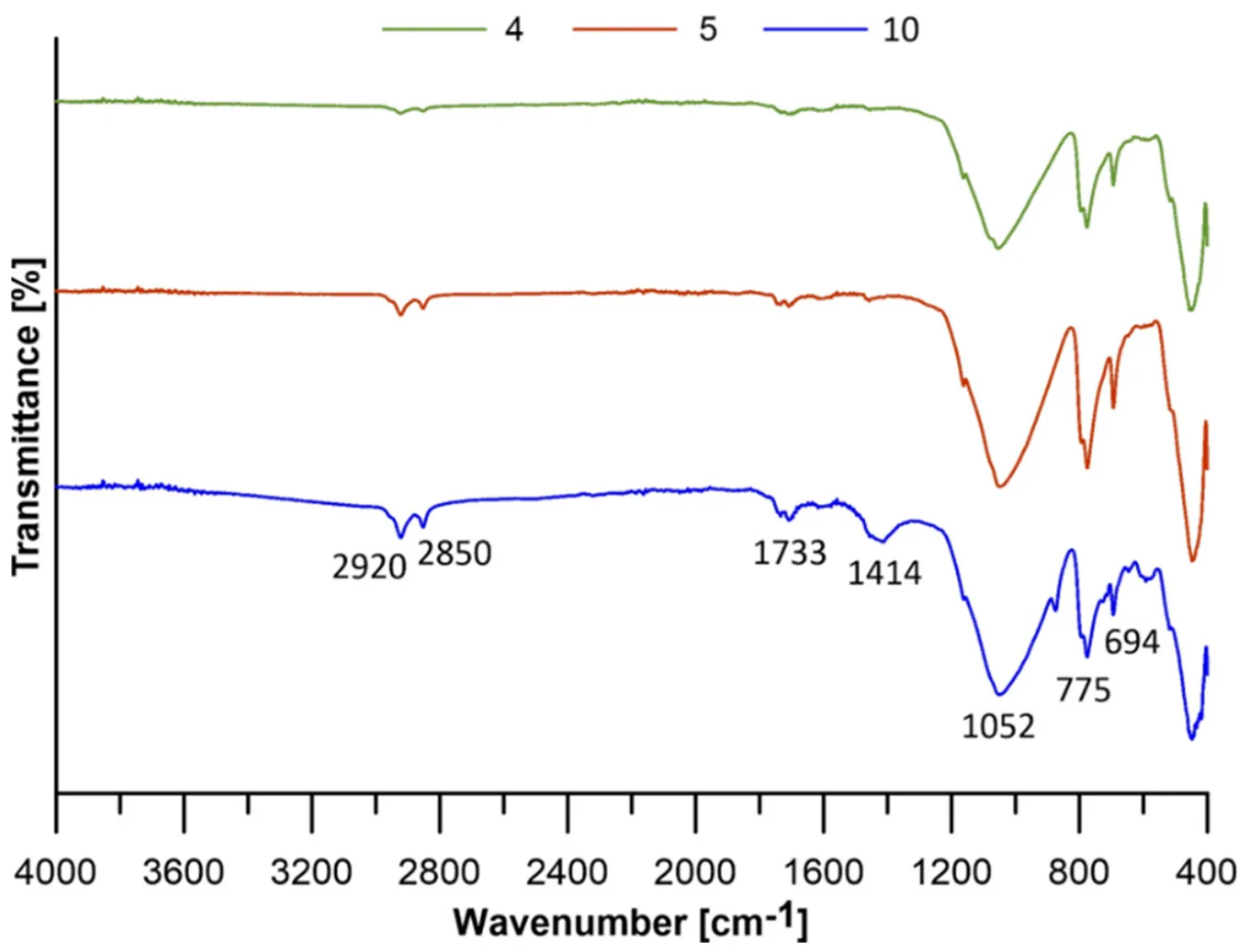
FT-IR spectra of composites 4, 5 and 10.

The ATR-FTIR spectra of selected turmeric-containing formulations are presented in Figure 3. Bands at approximately 2920 and 2850 cm^−1^ are consistent with asymmetric and symmetric CH_2_ stretching vibrations of lipid-derived aliphatic chains, while the band near 1733 cm^−1^ is assigned to ester carbonyl stretching. Because quartz is the major mineral component of the formulations, its contribution to the fingerprint region must be explicitly considered. The intense band/envelope in the approximately 1050–1100 cm^−1^ region, with a maximum near 1052 cm^−1^ in the present spectra, is assigned predominantly to Si-O stretching of the quartz phase, although overlap with C-O vibrations of organic constituents cannot be excluded. A feature near 1414 cm^−1^ is also observed; however, because the investigated cured formulations differ simultaneously in composition and processing conditions, no unique mechanistic interpretation is assigned to its variation.

Because the analysed samples differed simultaneously in formulation and curing conditions, and the relevant bands are not specific to a single chemical pathway, the spectra provide only indirect evidence of curing-associated chemical changes in the WCO-derived phase. Acid-assisted thermal curing may involve several concurrent transformations of the ester-containing and unsaturated lipid structures; however, the present FTIR results cannot distinguish among oxidation, oligomerisation, degradation and other possible reactions. The spectra do not by themselves demonstrate the formation of a crosslinked network or quantify crosslink density. Because quartz is the dominant mineral component of the investigated composites, the fingerprint region is interpreted primarily in relation to the mineral phase. The bands in the approximately 799–775 cm^−1^ region, the band near 694 cm^−1^, and the band at approximately 467 cm^−1^ are assigned predominantly to characteristic α-quartz vibrations and are therefore not used as markers of organic aromatic or torsional modes. The previous assignments of these features to C–H out-of-plane deformation, monosubstituted aromatic C–H bending, and organic torsional vibrations have been removed. The organic-phase interpretation is restricted primarily to the CH_2_ stretching bands near 2920 and 2850 cm^−1^ and the ester carbonyl band near 1733 cm^−1^, for which the assignments can be made with substantially greater confidence [23,24,25,26].

The ^1^H NMR spectrum of the acetone-soluble fraction extracted from the cured turmeric-containing WCO-based composite is presented in Figure S1. Because the analysis was performed on constituents extracted with deuterated acetone, the spectrum represents only the acetone-soluble fraction and not the insoluble cured binder phase as a whole. Signals at approximately 0.85, 1.27, 1.56 and 5.28 ppm are consistent with residual lipid-derived constituents, including terminal methyl, aliphatic methylene, carbonyl-adjacent methylene and olefinic proton environments, respectively [27,28]. The signal at approximately 2.05 ppm originated from acetone used during the extraction and measurement. Weak resonances in the aromatic region at approximately 7.10–7.15 ppm may be consistent with turmeric-derived or thermally transformed aromatic constituent [28]. However, signals in this region are not sufficiently specific to identify or quantify intact curcumin, particularly after prolonged curing at 190–210 °C. The spectrum presented in Figure S1 therefore confirms the presence of acetone-soluble lipid-derived and aromatic constituents but does not demonstrate the survival or retained concentration of intact curcumin after curing.

**Figure 4:**
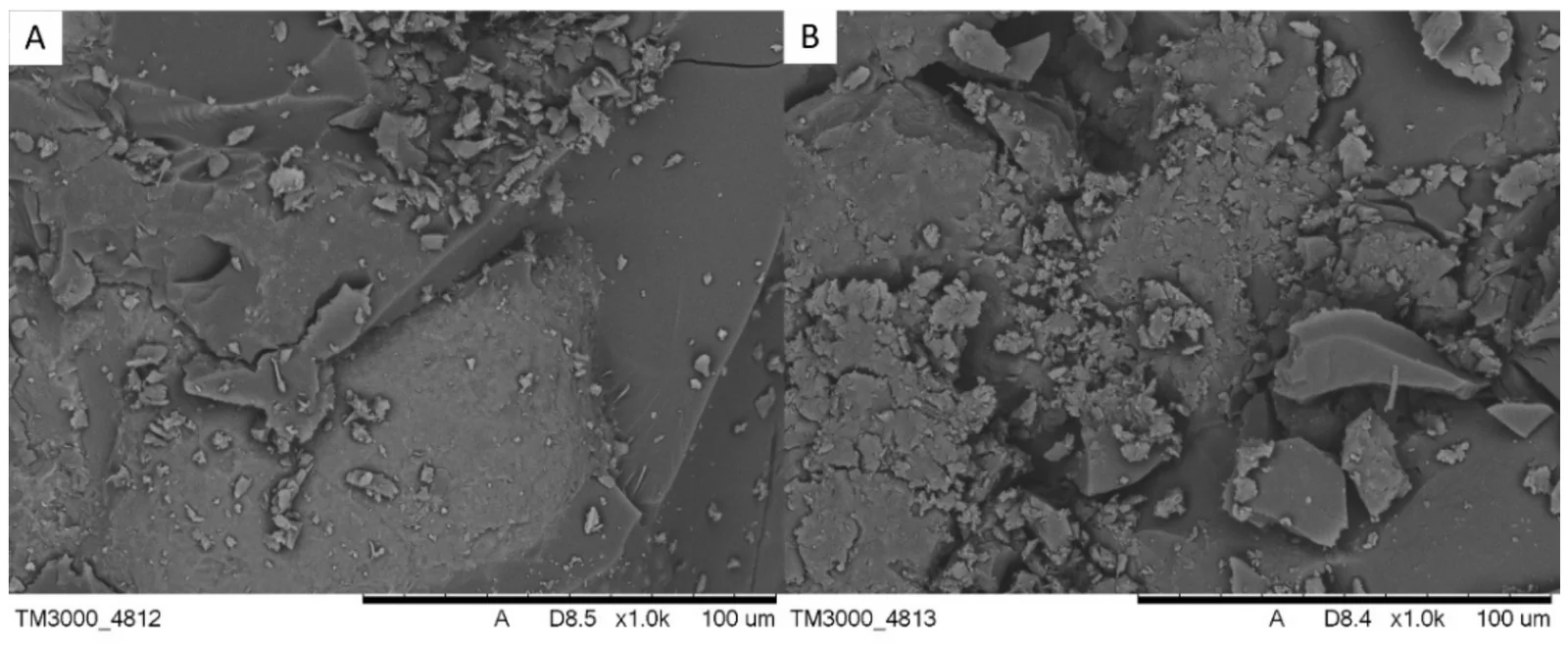
SEM micrographs of composites (**A**) 1 and (**B**) 4.

Figure 4 presents SEM micrographs of composites 1 and 4, while Figure S2 shows the corresponding EDS analysis of composite 4. EDS detected signals associated with the mineral fraction, carbon-containing constituents and sulfur-containing species. Palladium and gold signals originated from the conductive coating applied before observation. The sulfur signal confirms the presence of sulfur-containing material but does not establish the chemical form of sulfur or demonstrate the presence of free residual H_2_SO_4_.

The SEM images show a heterogeneous and porous microstructure, with regions in which the cured organic phase is visible around mineral particles and regions containing gaps or voids. Because only selected fields of view were examined and no quantitative porosity, interfacial-area or image-analysis measurements were performed, the micrographs are interpreted descriptively. Apparent differences in compaction, void frequency and particle embedding may be associated with the complete formulation and processing history. However, stronger matrix-filler adhesion, improved matrix continuity or more effective stress transfer cannot be established from the present images alone.

**Figure 5:**
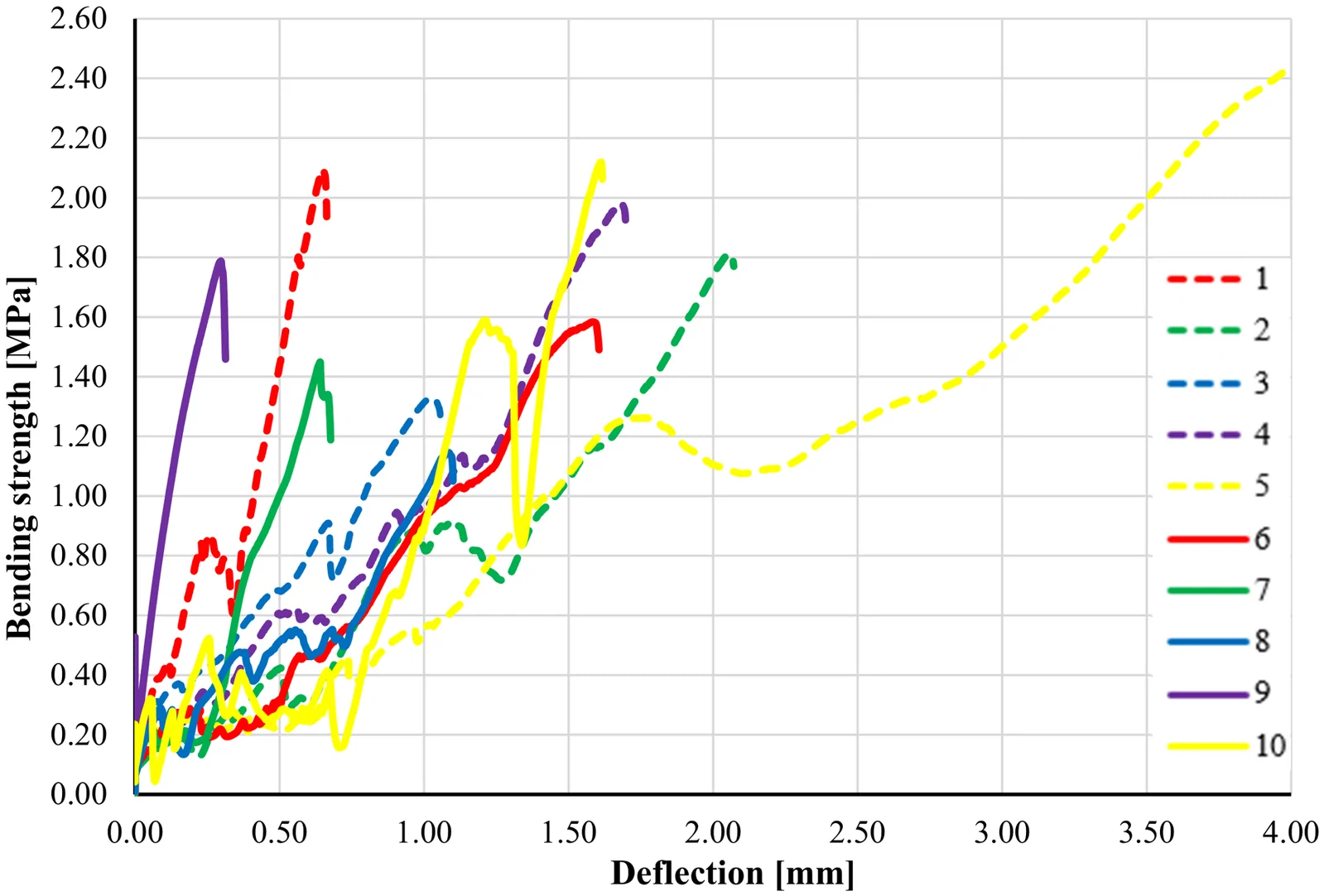
Bending stress-deflection curves for selected composite formulations.

Figure 5 presents bending stress-deflection curves for selected composite formulations. The measured bending strengths ranged from 1.15 to 2.42 MPa, whereas the splitting tensile strengths ranged from 0.30 to 0.52 MPa. Sample 5 exhibited the highest bending strength, while sample 4 exhibited the highest splitting tensile strength. The results demonstrate that the selected formulations formed self-supporting specimens with measurable mechanical integrity.

**Figure 6:**
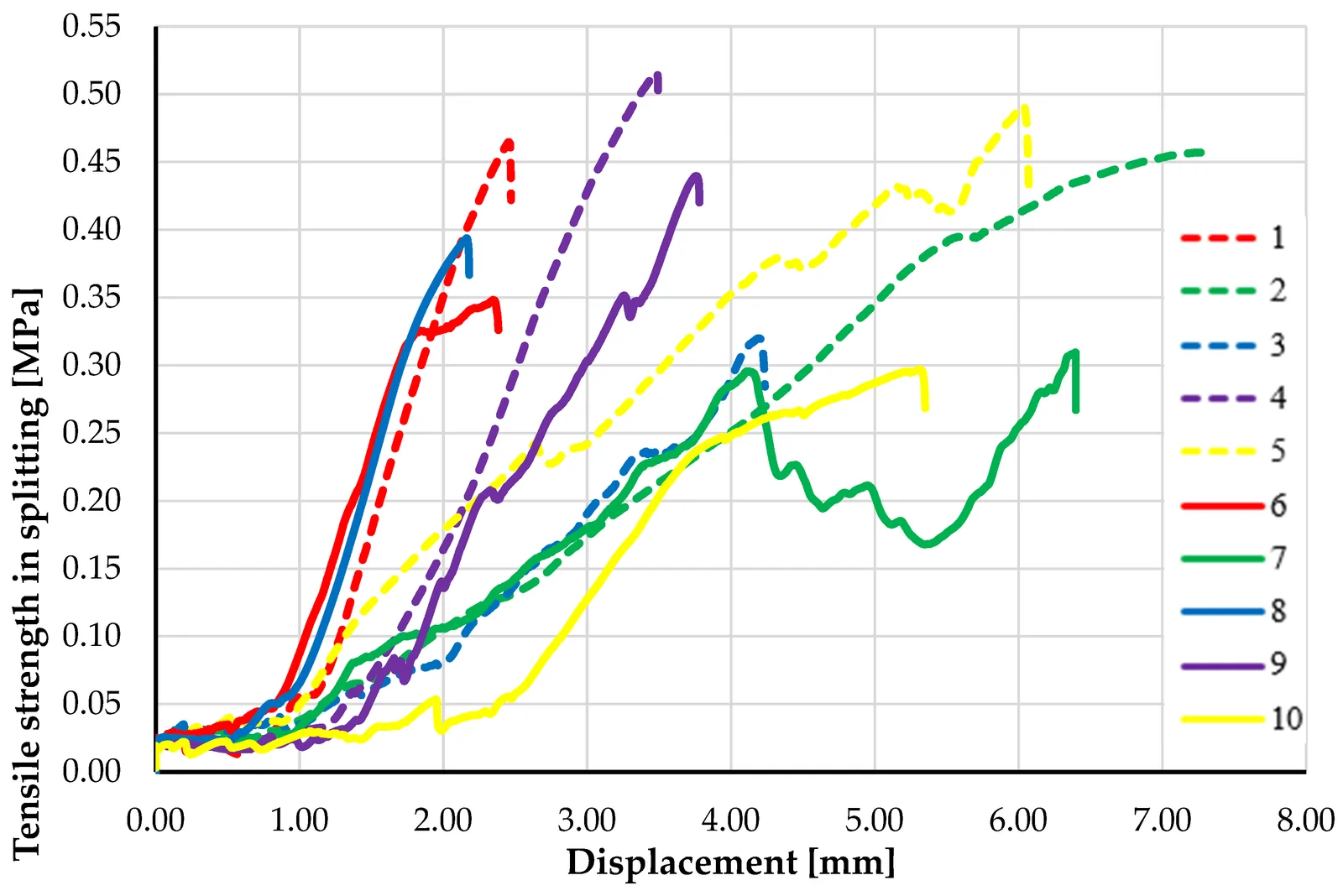
Splitting tensile stress-displacement curves for selected composite formulations.

Because catalysed-oil content, acid-to-oil ratio, turmeric content, curing temperature and curing time varied concurrently, the data do not isolate the independent effect of curing temperature, curing time or any other single factor. The differences among samples are therefore presented as formulation-level screening results rather than as evidence that one processing parameter caused the observed mechanical trend. The bending stress-deflection and splitting tensile stress-displacement curves for the selected composite formulations are presented in Figure 5 and Figure 6, respectively.

The displacement values and the shapes of the load–displacement curves also differed among formulations. Step-like changes visible in some curves are consistent with progressive damage during loading; however, the present measurements do not independently establish the sequence of microcracking, interfacial debonding or a crosslinking-controlled crack-arrest mechanism. Displacement at maximum load is therefore reported descriptively and is not used as a direct measure of elasticity, ductility or crack resistance.

Table 2 and Table 3 summarise the maximum bending strength and corresponding maximum deflection, as well as the maximum splitting tensile strength and corresponding displacement, for each formulation.

**Figure 7:**
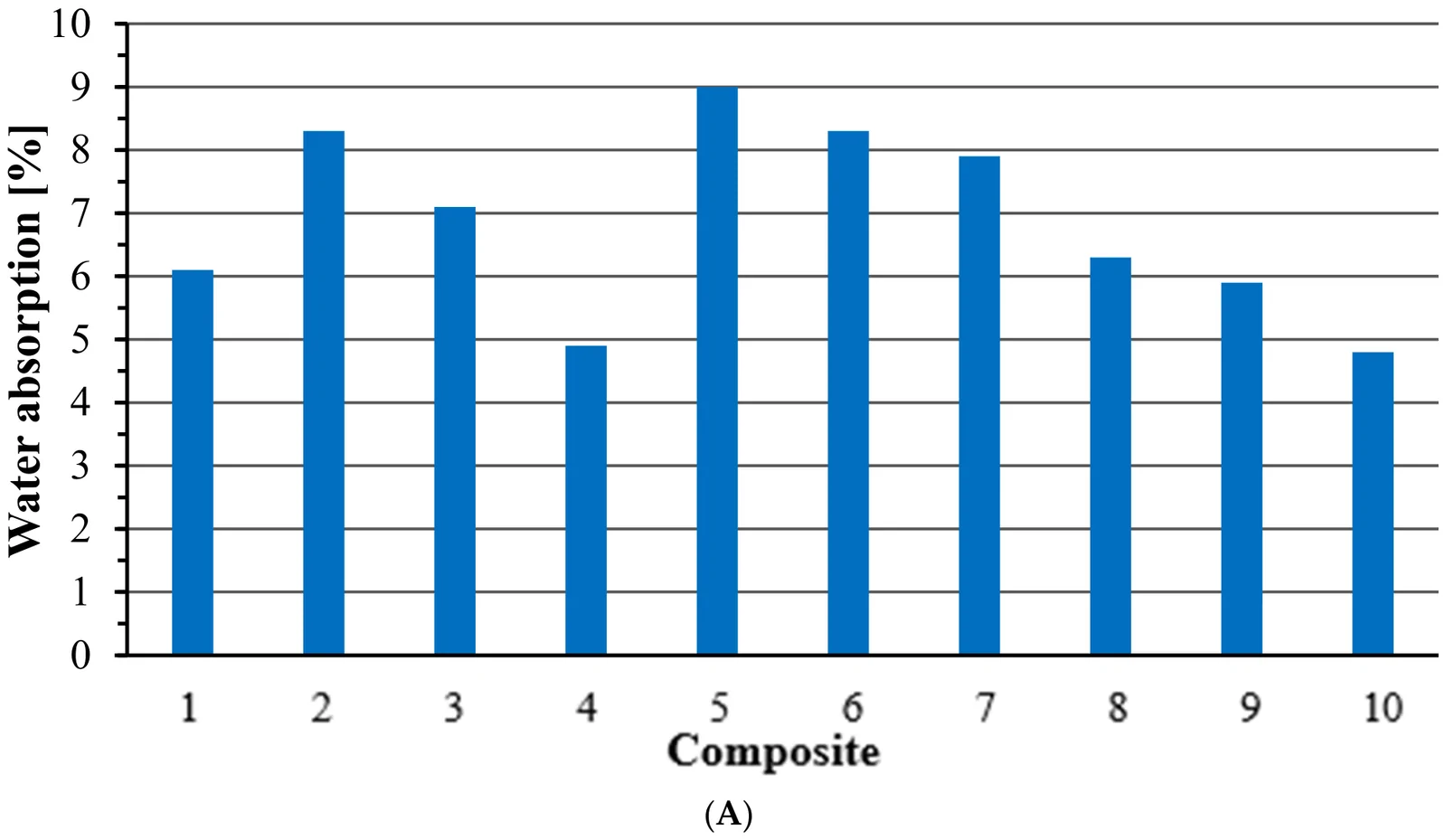

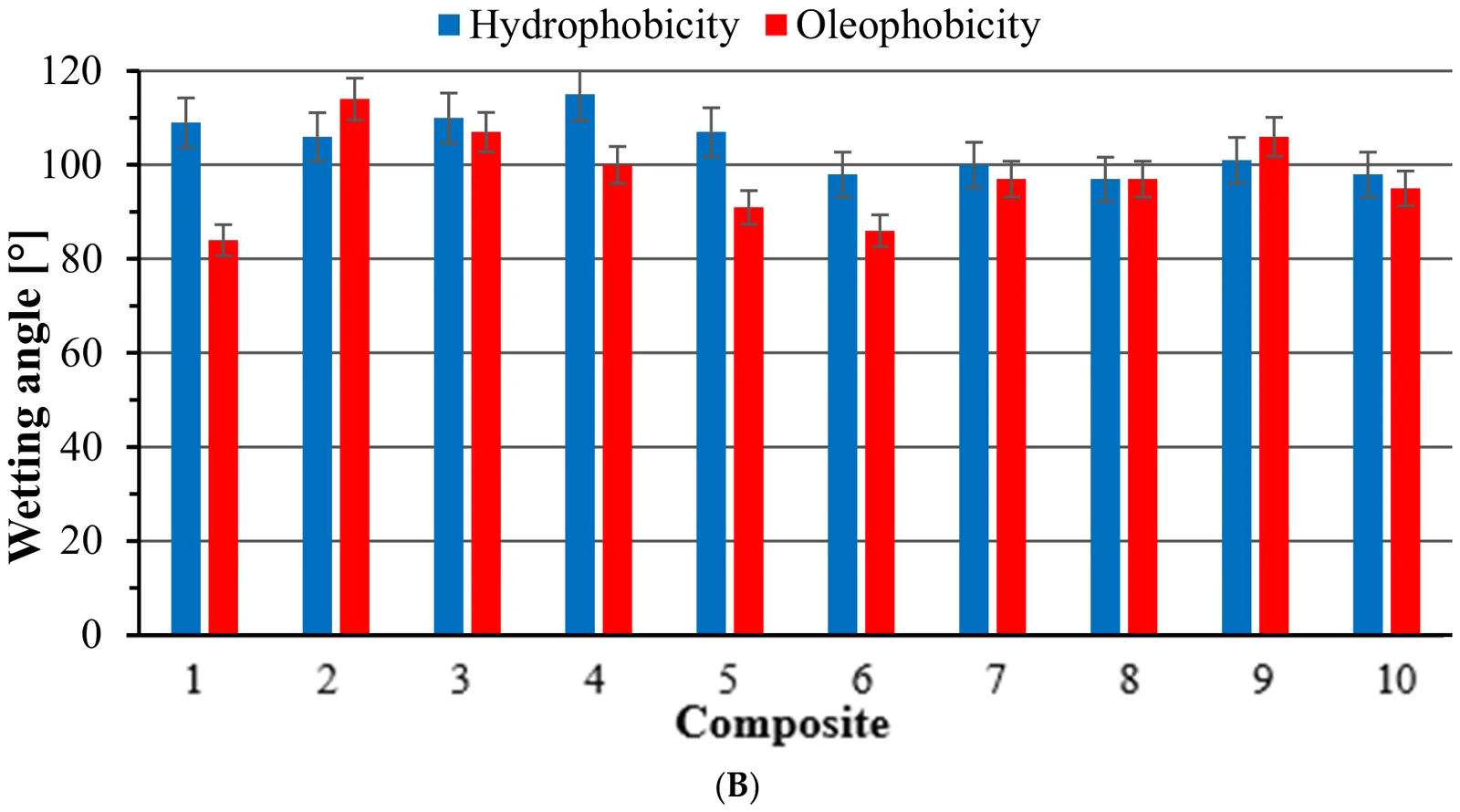
Water absorption (**A**) and static contact angles measured using deionised water and rapeseed oil (**B**) for turmeric-containing WCO-based polymer–mineral composites. Error bars in panels represent SD from 3 measurements.

The measured strength range confirms that the selected formulations formed self-supporting specimens. The values are substantially below those expected for structural load-bearing materials; therefore, the materials are classified here as experimental, non-load-bearing composites. The present mechanical screening does not by itself demonstrate suitability for a specific surface, construction or service application, which would require application-specific durability, adhesion, wear and ageing tests. Water absorption varied among the selected formulations, with the lowest measured value of approximately 4.8% for samples 4 and 10 (Figure 7A). This result reflects the total water uptake under the applied 72 h immersion procedure. Water absorption alone does not distinguish between open and closed porosity and cannot be used to quantify pore connectivity or crosslink density.

Water contact angles exceeded 90° for all tested formulations, indicating low surface wettability under the applied measurement conditions (Figure 7B). The contact-angle measurements characterise the interaction of the test liquid with the investigated surface but do not independently demonstrate reduced microbial adhesion or an antimicrobial mechanism. Samples 4 and 10 differed not only in curing time but also in formulation; therefore, their similar water absorption cannot be assigned specifically to curing duration.

### 3.2. Antimicrobial Performance of Turmeric-Containing WCO-Based Composites

The biological results are interpreted as an exploratory screening of complete turmeric-containing formulations. Because an otherwise identical turmeric-free composite and surface or eluate pH measurements were not available, the experimental design does not distinguish the contributions of turmeric-derived constituents, thermally transformed products, the cured WCO/acid-containing phase, residual acidity, surface wettability, porosity, or other formulation characteristics. In addition, because CFU enumeration was performed using aliquots recovered from the suspension after exposure, physical adhesion or sequestration of bacterial cells on or within the porous composite material may have contributed to the decrease in recoverable CFU. Material-associated cells were not separately recovered and enumerated, and therefore the relative contributions of physical sequestration and true loss of bacterial viability cannot be distinguished from the CFU data alone. Accordingly, the observed effects are attributed only to the complete tested formulations and are interpreted as reductions in recoverable viable cells under the applied assay conditions. No turmeric-specific, intact-curcumin-specific or predominantly contact-based antimicrobial mechanism is assigned. The surviving fractions determined after exposure to the composites are presented separately for bacterial and fungal microorganisms in the following subsections.

#### 3.2.1. Antibacterial Performance of Turmeric-Containing WCO-Based Composites

**Figure 8:**
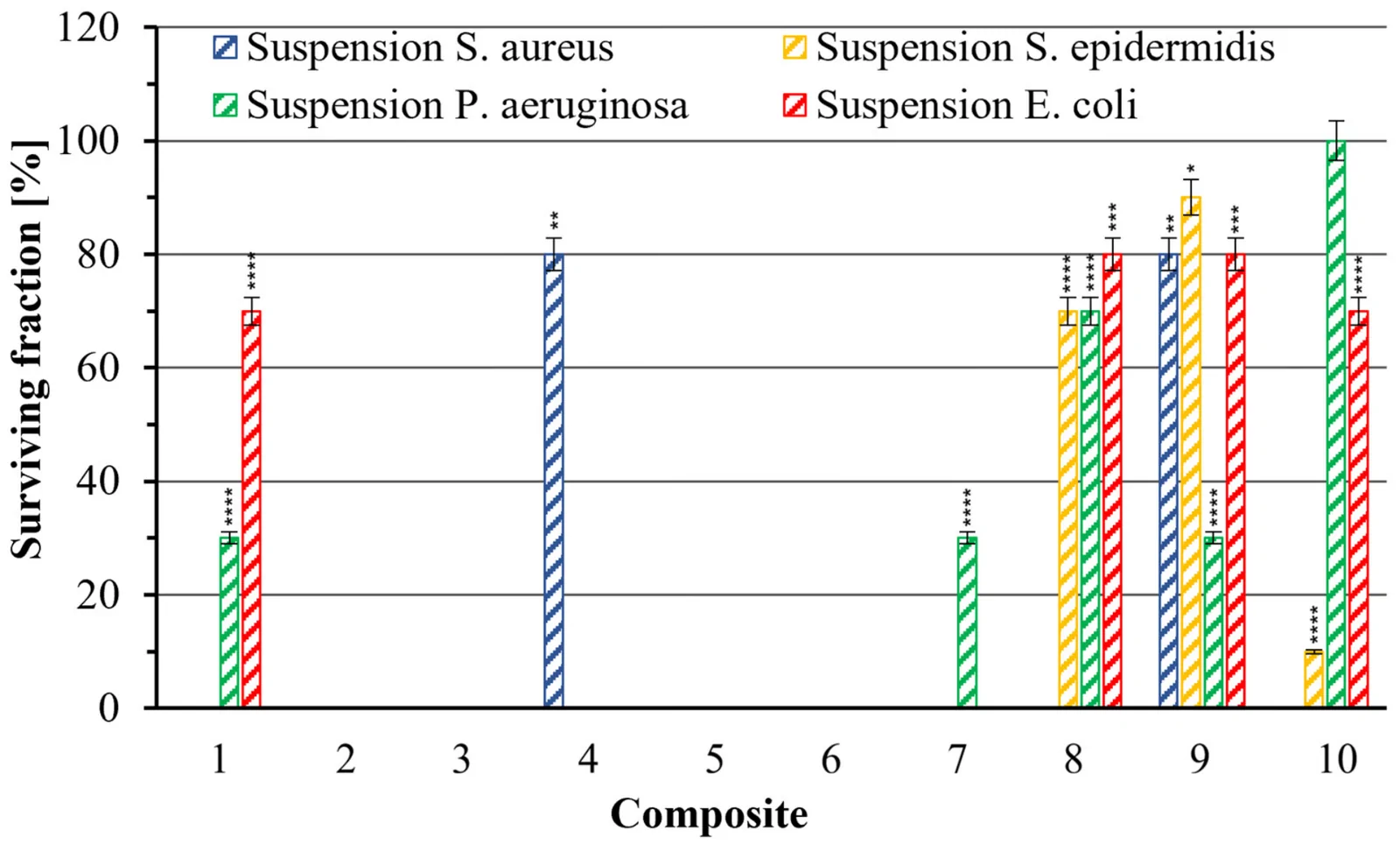
Surviving fractions of planktonic bacterial cultures after exposure to selected composite formulations at a nominal concentration of 100 mg mL^−1^. Surviving fractions are expressed relative to the corresponding untreated bacterial controls. Data are presented as mean values ± SEM (n = 3). Statistical significance was assessed separately for each bacterial species using one-way ANOVA followed by Dunnett’s multiple-comparison test against the corresponding untreated control. Statistical significance is indicated as follows: * *p* < 0.05, ** *p* < 0.01, *** *p* < 0.001, and **** *p* < 0.0001; the absence of an asterisk indicates *p* ≥ 0.05.

Figure 8 presents the surviving fractions of planktonic *S. aureus*, *S. epidermidis*, *E. coli* and *P. aeruginosa* after exposure to the tested formulations. Statistical comparison with the corresponding untreated controls confirmed significant reductions in surviving fraction for most formulation–bacterium combinations showing an apparent antimicrobial response. For *P. aeruginosa*, formulations 1, 7, 8 and 9 produced statistically significant reductions relative to the untreated control, whereas formulation 10 did not differ significantly from the control. The strongest responses were observed for formulations 1, 7 and 9, which reduced the surviving fraction to approximately 30%.

For *S. aureus*, formulations 4 and 9 reduced the surviving fraction by approximately 20%, and both reductions were statistically significant compared with the untreated control (*p* = 0.0041). Despite their statistical significance, these responses are interpreted as limited because of the relatively small magnitude of the reduction. For *S. epidermidis*, the strongest response was observed for formulation 10, which reduced the surviving fraction to approximately 10% (*p* < 0.0001). Formulations 8 and 9 also produced statistically significant, although less pronounced, reductions. All tested formulation-*E. coli* combinations showed statistically significant reductions relative to the untreated control.

No monotonic relationship between nominal turmeric content and antibacterial response was observed across the selected formulations. Because the formulations also differed in binder content, acid ratio and curing conditions, the statistical comparisons with the untreated controls demonstrate formulation-level biological responses but do not identify the contribution of turmeric or any other individual formulation variable. Differences among Gram-positive and Gram-negative species may be associated with their distinct cellular structures [29]; however, the active component and mechanism responsible for the observed responses were not determined.

**Figure 9:**
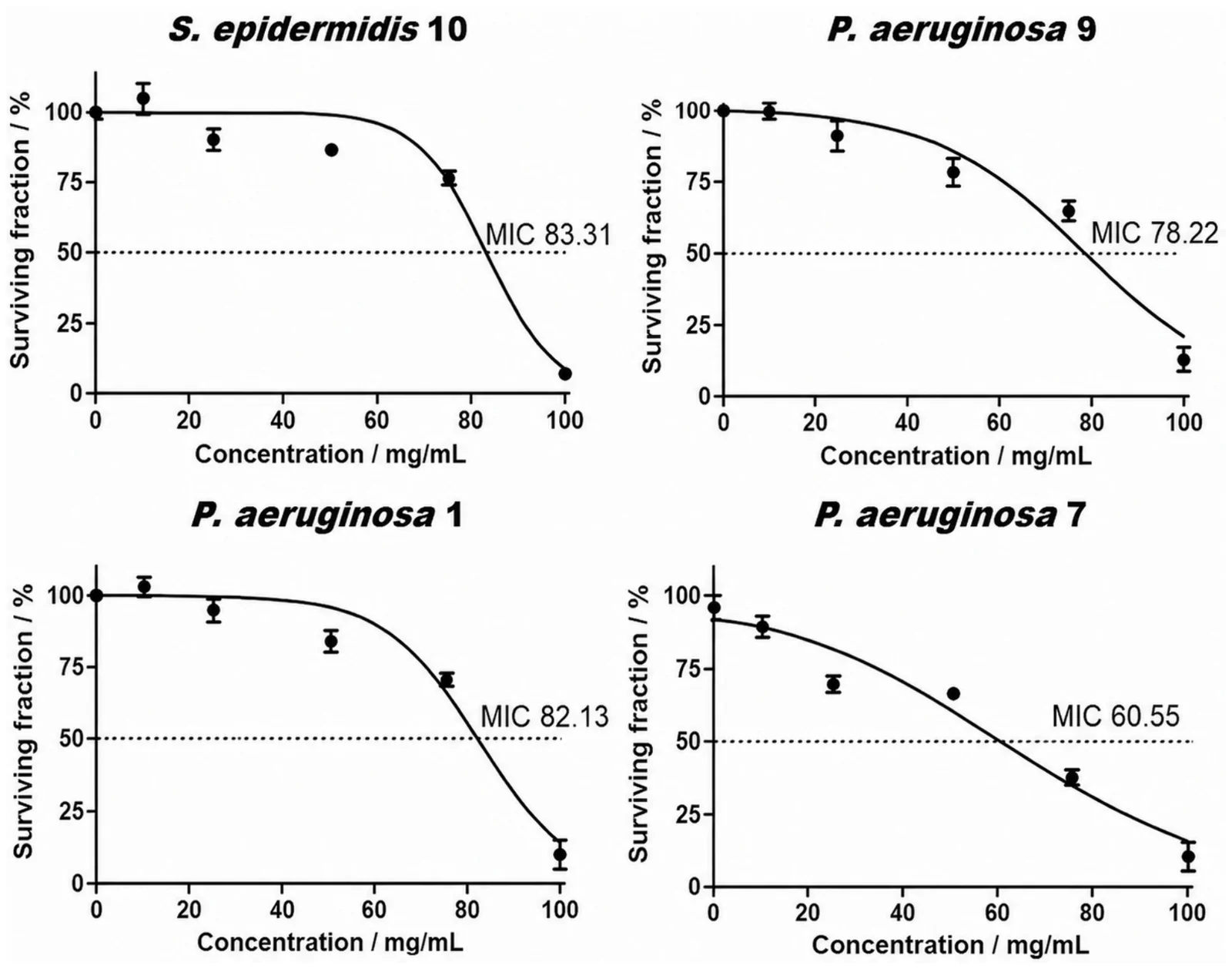
Concentration-response curves for selected formulation–bacterium combinations. Experimental surviving-fraction values are shown together with the nonlinear sigmoidal fits used to estimate the C_50_ values. The horizontal dotted line indicates a surviving fraction of 50%. Data points are presented as mean values ± SEM (n = 3).

Concentration-dependent measurements were subsequently performed for the selected formulation-organism combinations presented in Table 4 and Figure 9. The estimated C_50_ values ranged from approximately 60.6 to 83.3 mg mL^−1^, indicating measurable concentration-dependent responses at relatively high nominal composite concentrations. These values are interpreted as descriptive estimates obtained from empirical concentration-response fits and not as parameters for comparative pharmacodynamic modelling.

The quantitative antibacterial screening and concentration-response measurements were complemented by qualitative confocal-microscopy imaging of selected formulation–bacterium combinations.

**Figure 10:**
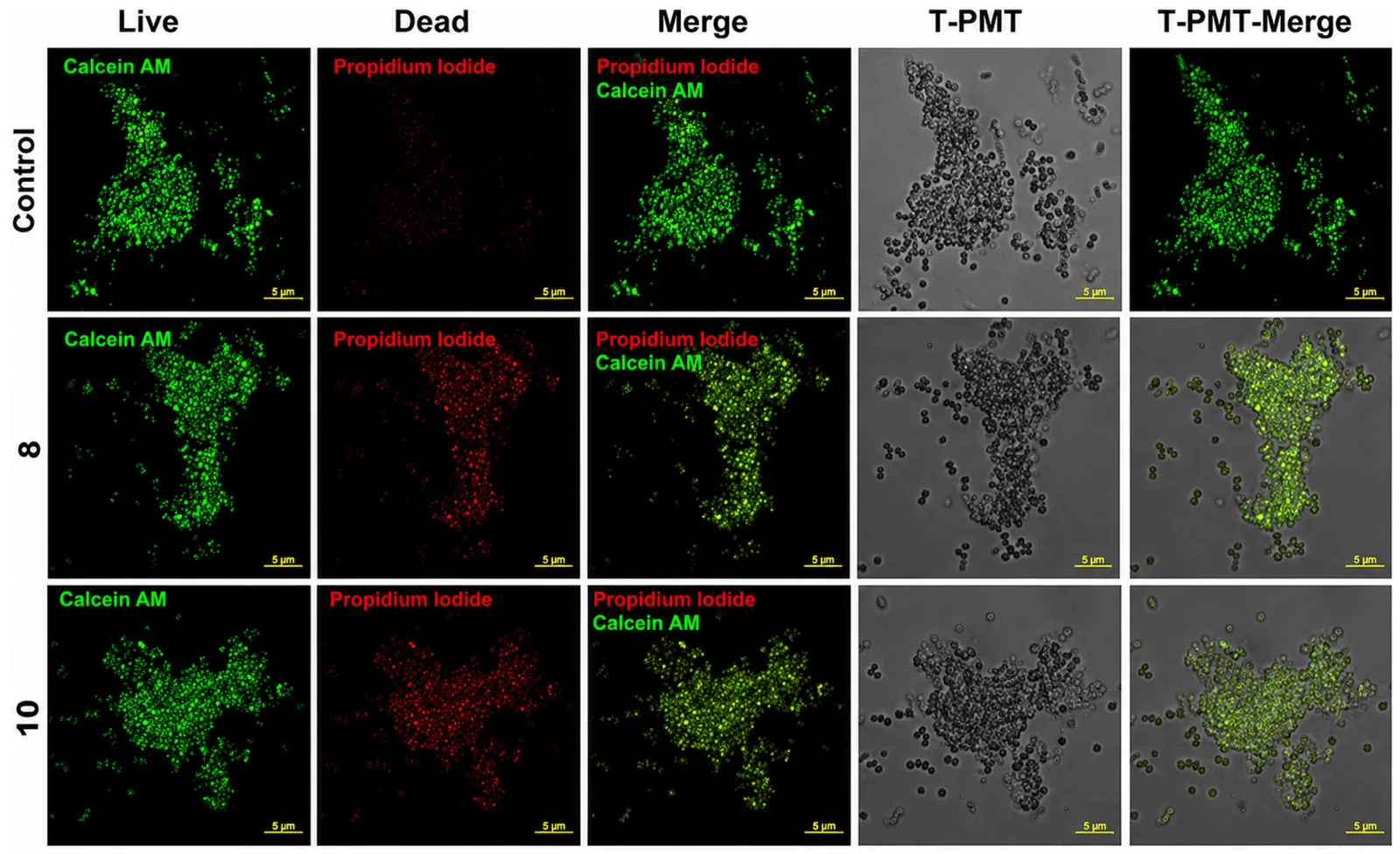
Representative confocal-microscopy images of *S. epidermidis* biofilms: untreated control and biofilms exposed to formulations 8 and 10.

**Figure 11:**
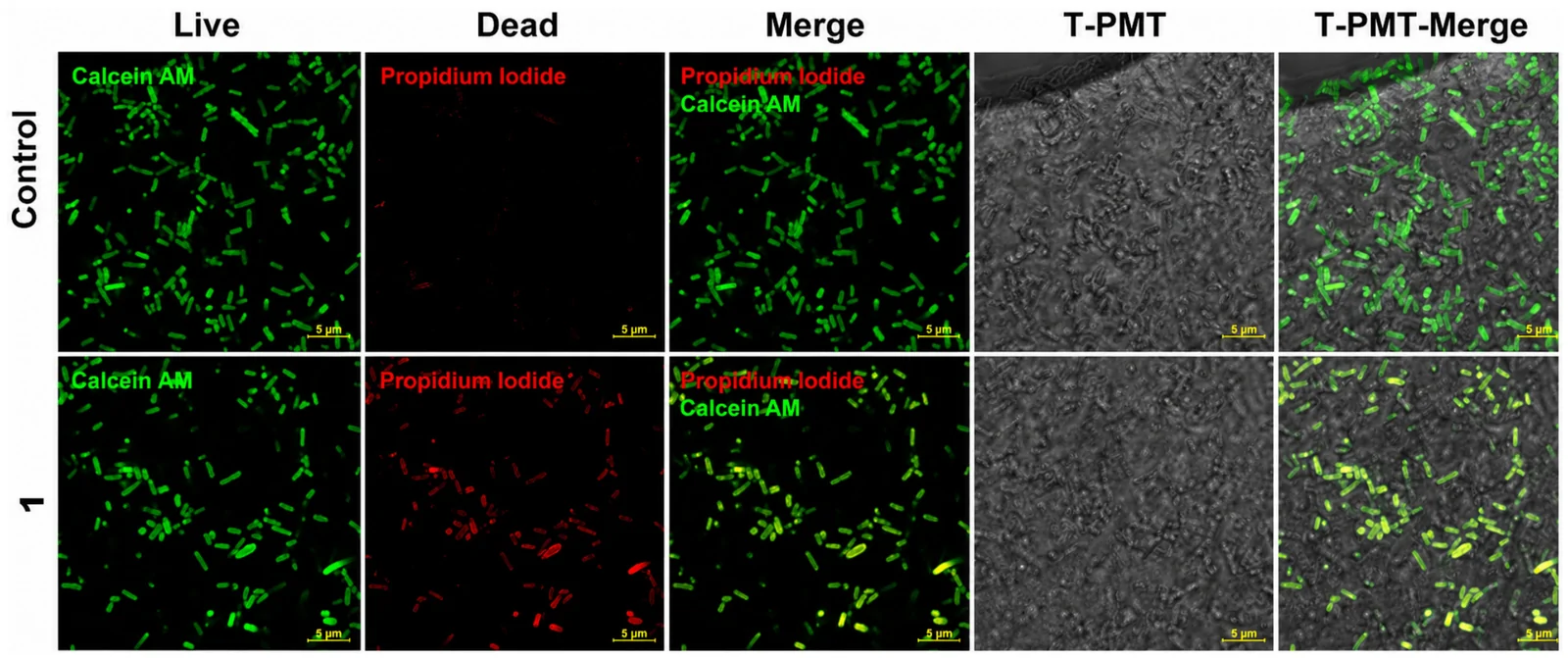
Representative confocal-microscopy images of *E. coli* biofilms: untreated control and biofilm exposed to formulation 1.

**Figure 12:**
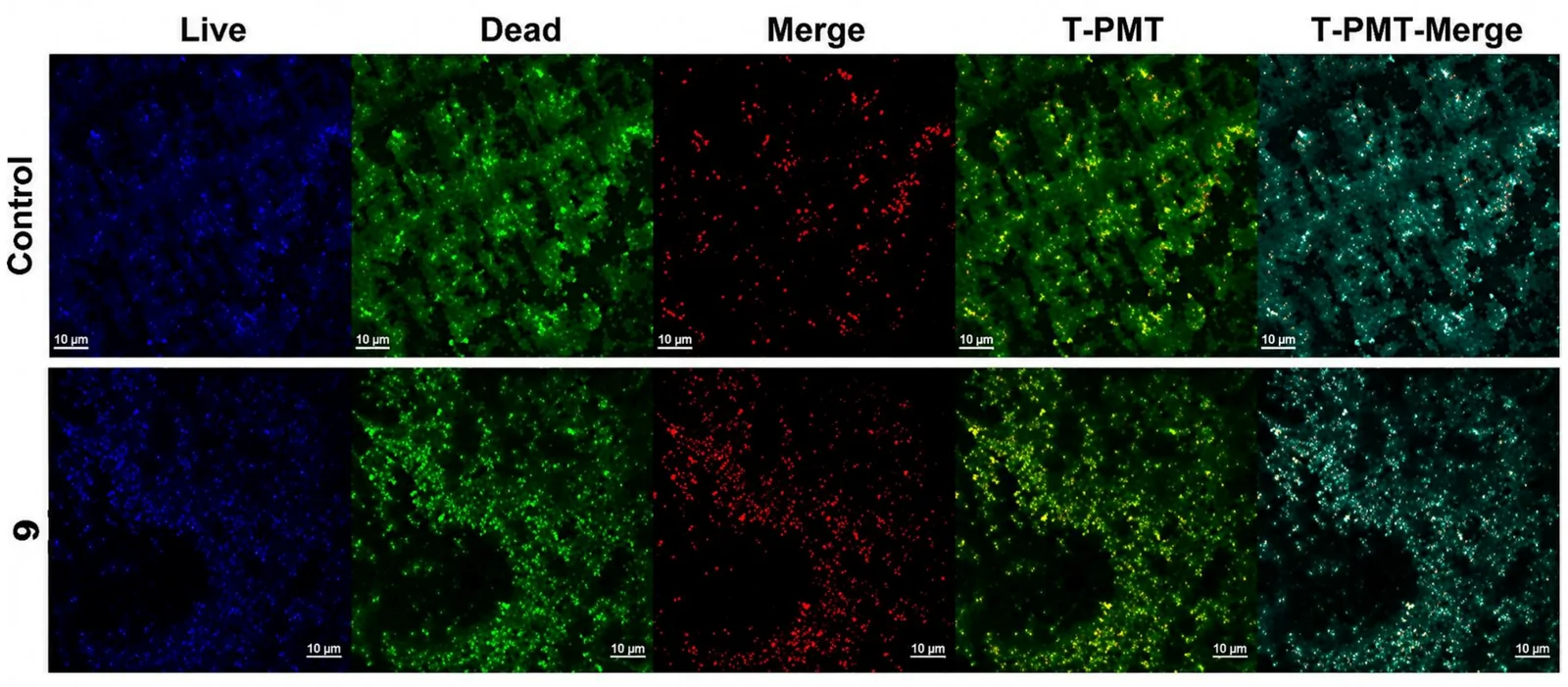
Representative confocal-microscopy images of *P. aeruginosa* biofilms: untreated control and biofilm exposed to formulation 9. Calcein AM and propidium iodide were used to visualise viable and membrane-damaged cells, respectively, whereas Hoechst dye was used for nucleoid staining. The images are presented as qualitative observations; no quantitative image analysis was performed.

Confocal microscopy was additionally used to visualise the response of selected bacterial biofilms after exposure to the composite formulations. Representative fluorescence images obtained for *S. epidermidis*, *E. coli*, and *P. aeruginosa* are presented in Figure 10, Figure 11 and Figure 12, respectively. These images illustrate the distribution of Calcein AM- and PI-positive cells within the examined biofilms and are interpreted as qualitative complementary observations. The *P. aeruginosa* images obtained after exposure to formulation 9 provide an additional qualitative view of the response of this organism, which was also represented in the suspension-based screening and concentration-response measurements. Importantly, the confocal biofilm experiments and the suspension-based CFU assay represent different biological states and experimental readouts. Therefore, the confocal images are not used to derive numerical surviving fractions or as quantitative validation of the CFU-based results.

#### 3.2.2. Antifungal Performance of Turmeric-Containing WCO-Based Composites

**Figure 13:**
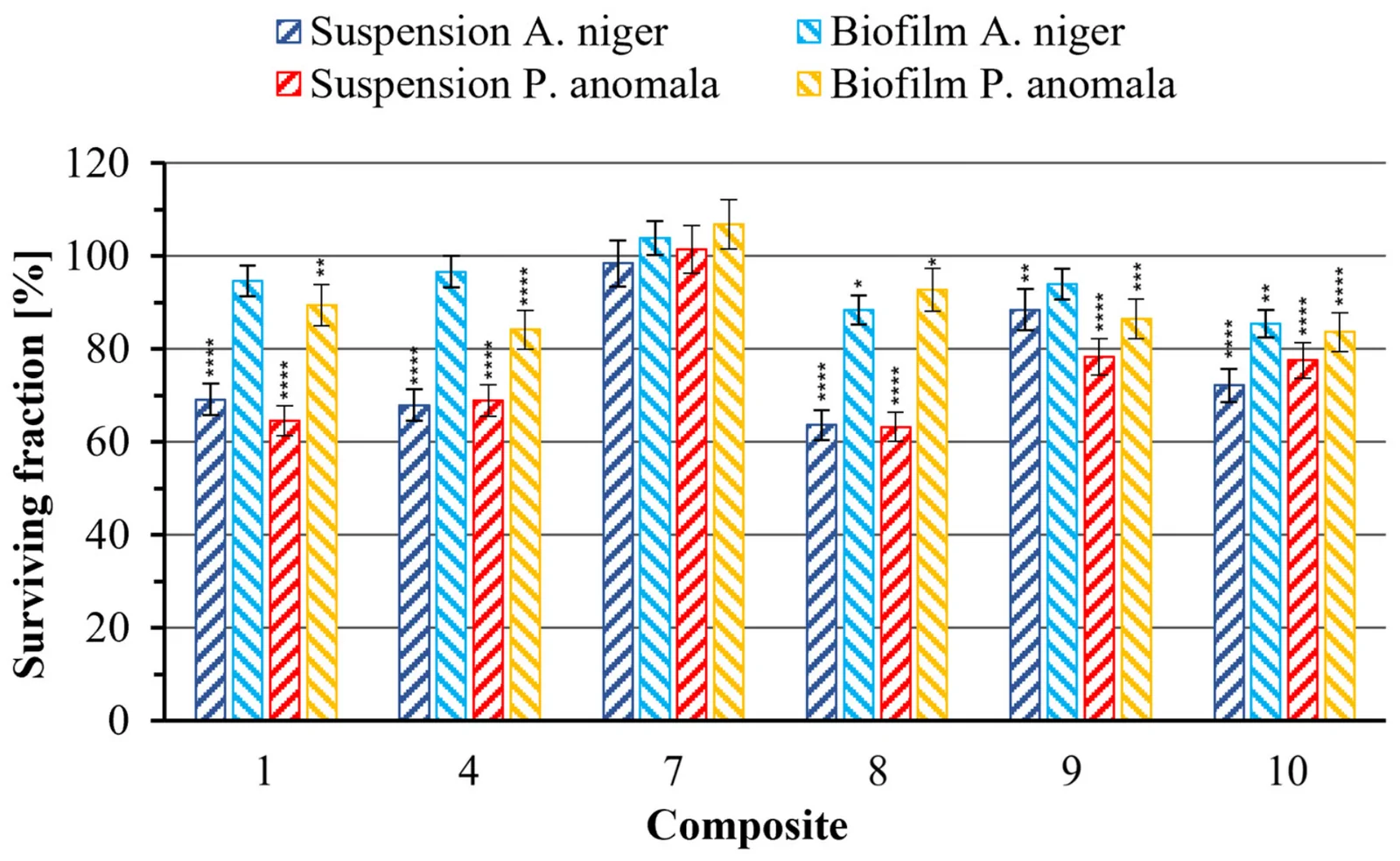
Surviving fractions of planktonic and biofilm forms of *A. niger* and *P. anomala* after 24 h exposure to selected composite formulations at a nominal concentration of 100 mg mL^−1^. Surviving fractions are expressed relative to the corresponding untreated microorganism controls. Data are presented as mean values ± SEM (n = 3). Statistical significance was assessed separately for each microorganism and culture form using one-way ANOVA followed by Dunnett’s multiple-comparison test against the corresponding untreated control. Statistical significance is indicated as follows: * *p* < 0.05, ** *p* < 0.01, *** *p* < 0.001, and **** *p* < 0.0001; the absence of an asterisk indicates *p* ≥ 0.05.

The antifungal screening revealed formulation- and culture-form-dependent responses of *A. niger* and *P. anomala* (Figure 13). Statistical comparison with the corresponding untreated controls confirmed that the responses were generally more pronounced for planktonic cultures than for the corresponding biofilms [30]. For planktonic *A. niger*, formulations 1, 4, 8, 9 and 10 produced statistically significant reductions in surviving fraction relative to the untreated control, whereas formulation 7 showed no significant effect. The lowest surviving fractions were observed for 8, 4 and 1. In contrast, for *A. niger* biofilms, significant reductions were detected only for formulations 8 and 10, while 1, 4, 7 and 9 did not differ significantly from the untreated control. This result supports the generally weaker response of the *A. niger* biofilm under the applied assay conditions. For planktonic *P. anomala*, all tested formulations except 7 produced statistically significant reductions relative to the untreated control, with the strongest responses observed for 1, 4 and 8. For *P. anomala* biofilms, statistically significant reductions were observed for formulations 1, 4, 8, 9 and 10, whereas 7 did not differ significantly from the untreated control. Thus, formulation 7 consistently showed weak or negligible antifungal effects, with surviving fractions approaching or exceeding 100%. Although several statistically significant differences were detected, the magnitude of the biological response varied considerably among formulations and between planktonic and biofilm cultures. Because the formulations differed simultaneously in composition and curing conditions and no matched turmeric-free composite was included, these differences cannot be attributed independently to turmeric content, intact curcumin or a specific formulation variable and are therefore interpreted as formulation-level screening responses.

### 3.3. Integrated Interpretation, Significance and Study Scope

The present study demonstrates that waste cooking oil can be converted into a cured organic binder capable of consolidating quartz-filled, turmeric-containing composite specimens. All selected formulations formed self-supporting materials with measurable mechanical integrity, while the combined mechanical, water-absorption and wettability results confirmed substantial formulation-dependent differences. The highest bending and splitting tensile strengths reached 2.42 and 0.52 MPa, respectively, the minimum water absorption was approximately 4.8%, and all investigated surfaces exhibited water contact angles above 90°. These findings indicate that the developed materials combine mechanical cohesion with limited water uptake and low surface wettability.

Although the obtained strength values are below those required for structural load-bearing materials, they are relevant to the development of experimental non-load-bearing and mineral-filled composite systems. Previous studies have demonstrated the potential of WCO-containing materials in construction-related and other non-conventional composite applications [5,16]. The present study extends this research direction by combining physicochemical, mechanical, surface and biological characterisation within a single group of waste-derived composite formulations.

The spectroscopic, thermal and microscopic analyses provide complementary, method-specific characterisation of the cured WCO-derived binder/mineral system. FTIR identifies lipid-derived functional groups together with prominent quartz-related bands, while SEM-EDS shows a heterogeneous mineral-rich structure containing an organic phase around quartz particles. For formulation 1, TGA revealed thermally induced mass-loss events under nitrogen, including a principal mass-loss region attributed broadly to decomposition of the cured organic fraction. However, because the small TGA analytical portion may not be representative of the bulk composite, the residue measured at 900 °C is not used for quantitative compositional interpretation and the TGA result is not generalised to the other formulations. Accordingly, formulation-level comparisons throughout this study refer to the nominal pre-curing inputs and processing conditions; post-curing component mass fractions were not determined. Considered together, these results support the formation of a solid cured binder system, while avoiding quantitative claims regarding crosslink density that were outside the analytical scope of the present study.

The microbiological screening revealed clear formulation- and microorganism-dependent responses. Selected formulations produced pronounced reductions in the recoverable CFU-derived surviving fractions of *S. epidermidis* and *P. aeruginosa*. However, because the suspension assay did not include an inert particulate control or separate recovery and enumeration of cells associated with the composite material, physical adhesion or sequestration may have contributed to the observed reduction in recoverable CFU. The qualitative confocal-microscopy images provide complementary visualisation of membrane-damaged and viable cells in selected exposed biofilms; however, they do not quantify the fraction of cells retained on the material and therefore do not exclude physical sequestration as a contributing factor. The weaker activity observed for some formulations and for fungal biofilms demonstrates that biological performance cannot be predicted solely from the nominal turmeric content and is instead associated with the complete composition and processing history of the material. This formulation-level variability constitutes an important outcome of the screening because it identifies compositions with the most promising balance of physical and biological properties for subsequent targeted development.

The study was designed as an exploratory multifactorial screening of selected formulations rather than as a factorial analysis of individual formulation variables. Consequently, the results establish differences among complete composite systems but do not isolate the independent contribution of curing temperature, curing time, binder content, acid ratio or turmeric concentration. Similarly, because the biological response was evaluated for complete turmeric-containing formulations, the present results are not used to assign the observed activity exclusively to intact curcumin or to a single antimicrobial mechanism. This interpretation does not reduce the validity of the measured responses but defines the level at which conclusions can be drawn from the current experimental design.

Overall, the results provide a coherent physicochemical and biological characterisation of a new group of turmeric-containing WCO-based mineral composites. They demonstrate the feasibility of obtaining materials combining low surface wettability, mechanical cohesion and formulation-dependent biological responses under the applied screening conditions from a waste-derived oil precursor and identify promising formulations for further optimisation. Future studies may focus on the most active compositions and employ matched formulation controls, an inert particulate control, and separate recovery and enumeration of material-associated cells to distinguish physical sequestration from loss of microbial viability. Targeted chemical analyses may additionally clarify the contribution of individual formulation components and support assessment of long-term performance under application-relevant conditions.

## 4. Conclusions

This study demonstrates the feasibility of using post-consumer waste cooking oil as a precursor of a cured organic binder capable of consolidating quartz-filled, turmeric-containing composite specimens. The selected formulations formed self-supporting materials with bending strengths of 1.15–2.42 MPa and splitting tensile strengths of 0.30–0.52 MPa. The minimum measured water absorption was approximately 4.8%, while water contact angles exceeded 90° for all investigated formulations, indicating low surface wettability under the applied conditions. The combined FTIR, ^1^H NMR analysis of acetone-soluble constituents and SEM-EDS results characterise the cured mineral-rich composites and support the presence of a solid WCO-derived binder phase. TGA performed for formulation 1 showed thermally induced mass-loss events under nitrogen; however, because the small analytical portion may not be representative of the bulk composite, the measured residue was not used to determine retained organic content or post-curing composition and was not generalised to the other formulations. The present results also do not quantify crosslink density. The microbiological screening revealed formulation- and microorganism-dependent responses. Selected formulations produced pronounced reductions in the surviving fractions of *S. epidermidis* and *P. aeruginosa*, whereas the responses of fungal biofilms were more limited. The C_50_ values show that measurable concentration-dependent responses were obtained at relatively high nominal composite concentrations. Because the selected formulations differed simultaneously in several composition and processing parameters and no matched turmeric-free composite was included, the observed biological effects are attributed to the complete composite formulations rather than exclusively to turmeric, intact curcumin or a single antimicrobial mechanism. Overall, the study provides an integrated physicochemical and biological characterisation of a new group of turmeric-containing WCO-based mineral composites and identifies formulations combining mechanical cohesion, limited water uptake, low surface wettability and measurable antimicrobial response under the applied screening conditions. The results provide a basis for the targeted development of non-load-bearing functional composite materials from a waste-derived oil precursor.

## Figures and Tables

**Table 1:** Formulation and thermal-curing conditions of the selected turmeric-containing WCO-based composites.

Composite No.	Catalysed Oil [%]	H_2_SO_4_/Catalysed Oil [g/g]	Temperature [°C]	Time [h]	Turmeric Content [%]
1	25	0.24	190	20	1
2	25	0.24	210	20	7
3	20	0.24	210	18	7
4	25	0.24	210	18	1
5	20	0.24	210	20	1
6	22.5	0.14	200	18	4
7	22.5	0.24	200	19	4
8	22.5	0.24	190	19	1
9	22.5	0.24	210	19	1
10	22.5	0.24	210	12	1

**Table 2:** Bending strength of turmeric-containing WCO-based polymer–mineral composites.

No.	Bending Strength [MPa]	Deviation from the Mean [MPa]	Deflection [mm]	Deviation from the Mean [mm]
1	2.10	0.327	0.65	−0.81
2	1.81	0.037	2.05	0.59
3	1.32	−0.453	1.03	−0.43
4	1.98	0.207	1.70	0.24
5	2.42	0.647	3.98	2.52
6	1.59	−0.183	1.59	0.13
7	1.45	−0.323	0.64	−0.82
8	1.15	−0.623	1.10	−0.36
9	1.79	0.017	0.30	−1.16
10	2.12	0.347	1.60	0.14
Mean value	1.77		1.46	
Standard deviation	0.397		1.04	

**Table 3:** Splitting tensile strength of turmeric-containing WCO-based polymer–mineral composites.

No.	Tensile Strength in Splitting [MPa]	Deviation from the Mean [MPa]	Displacement [mm]	Deviation from the Mean [mm]
1	0.46	0.055	2.45	−1.90
2	0.47	0.065	7.25	2.90
3	0.32	−0.085	4.25	−0.10
4	0.52	0.115	3.50	−0.85
5	0.49	0.085	6.00	1.65
6	0.35	−0.055	2.35	−2.00
7	0.31	−0.095	6.40	2.05
8	0.39	−0.015	2.20	−2.15
9	0.44	0.035	3.75	−0.60
10	0.30	−0.105	5.35	1.00
Mean value	0.405		4.35	
Standard deviation	0.083		1.81	

**Table 4:** Estimated C_50_ values for selected formulation–bacterium combinations.

Strain	Material	C_50_ (mg/mL)
*S. epidermidis*	10	83.3
*P. aeruginosa*	9	78.2
*P. aeruginosa*	1	82.1
*P. aeruginosa*	7	60.6

## Data Availability

The original contributions presented in this study are included in the article. Further inquiries can be directed to the corresponding author.

## Supplementary Materials

The following supporting information can be downloaded at: https://www.mdpi.com/article/10.3390/ma19173583/s1, Figure S1: ^1^H NMR spectra of composite 1 (above) and composite 8 (below); Figure S2: EDS analysis and elemental content chart of composites 4.
