# Supplementary material for: Turmeric-Containing Polymer–Mineral Composites with a Waste Cooking Oil-Derived Binder: Physicochemical Characterisation and Exploratory Antimicrobial Screening
Source: Materials (Basel). 2026 Aug 24;19(17):3583. doi: 10.3390/ma19173583 (PMC13566464; doi:10.3390/ma19173583)
Supplement: Supplementary file 1 [file materials-19-03583-s001.zip › materials-4431396-supplementary.pdf]

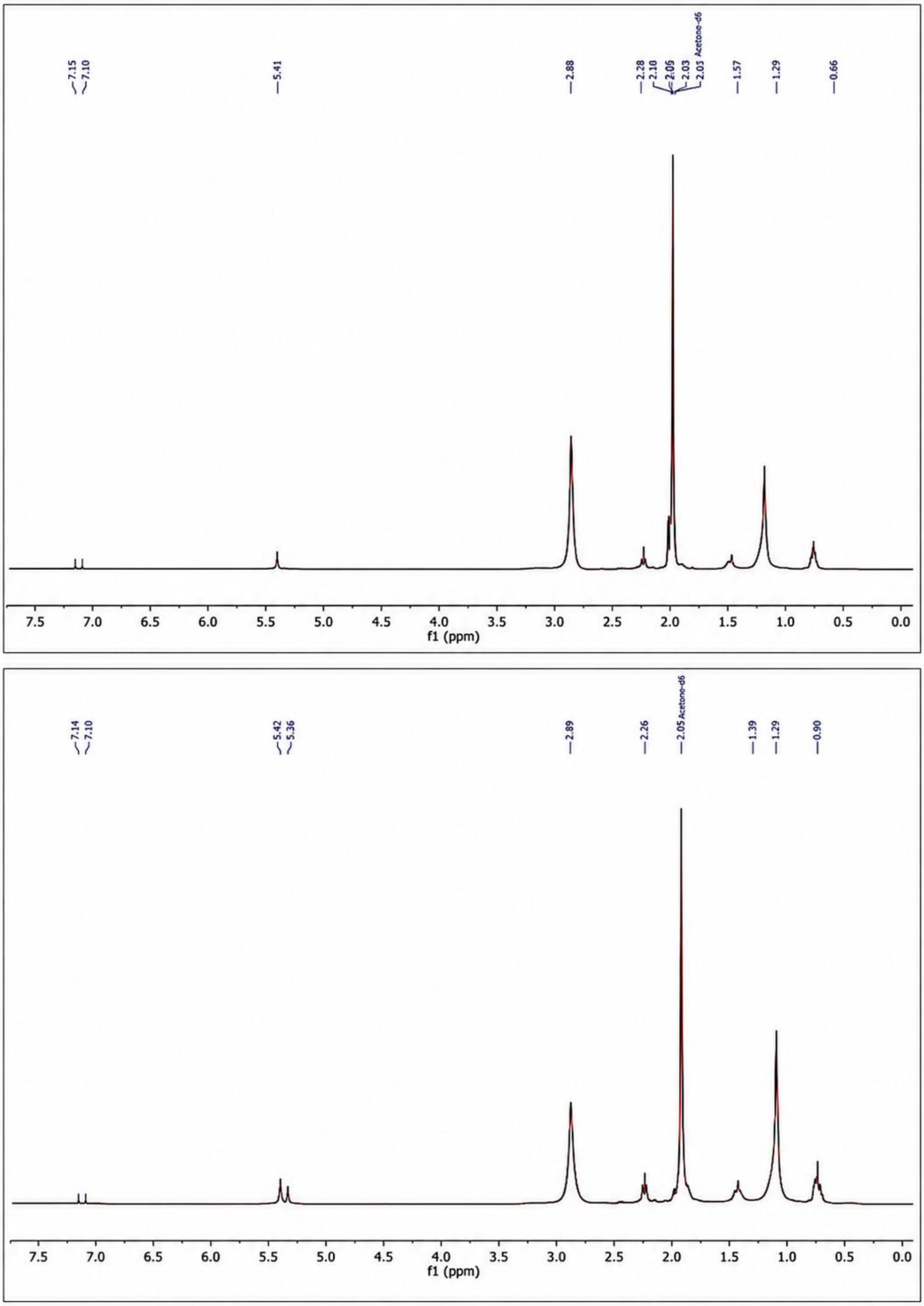

Figure S1. ^{1}H NMR spectra of composite 1 (above) and composite 8 (below).

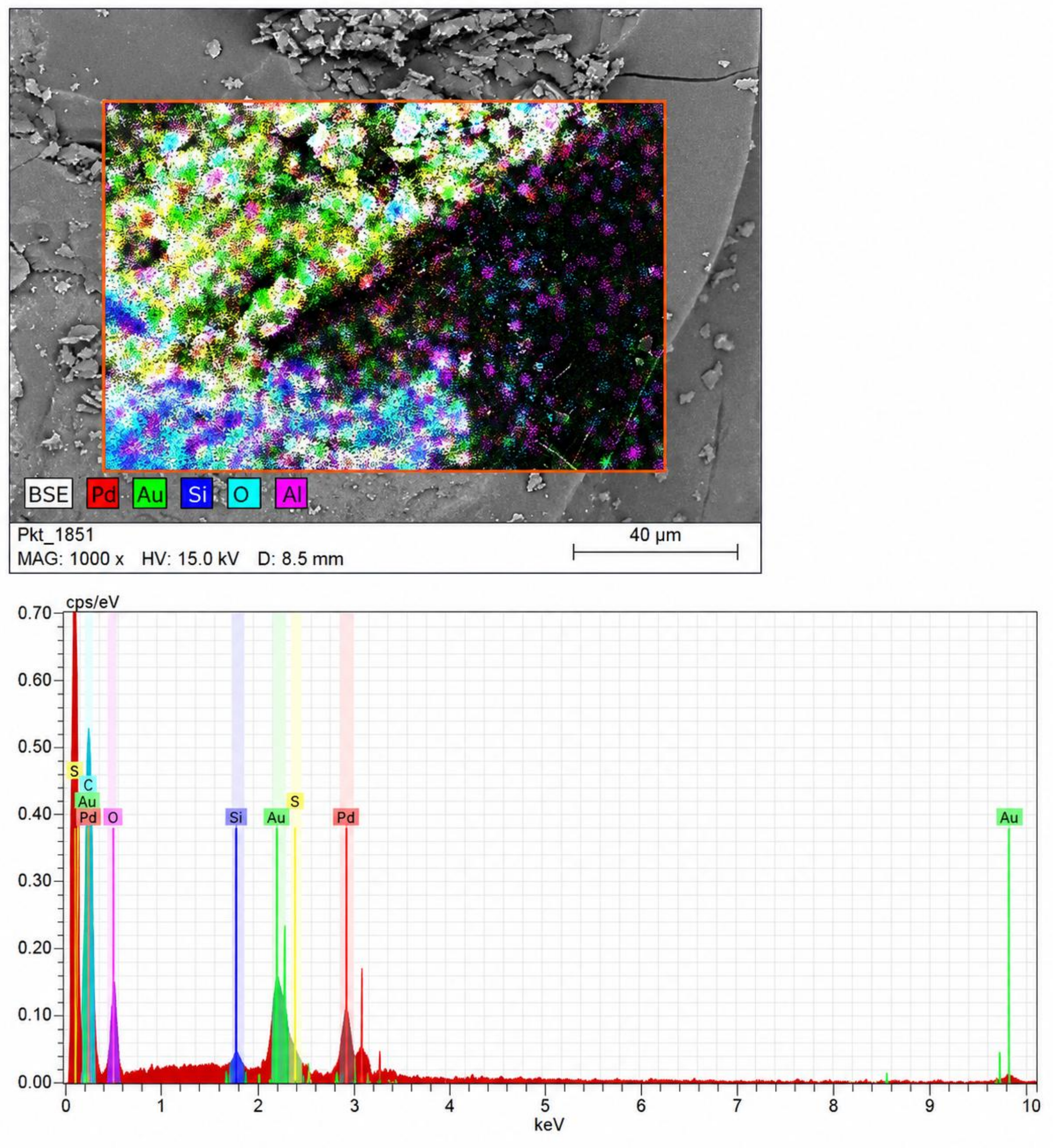

Figure S2. EDS analysis and elemental content chart of composites 4.
